# Hide and Seek: Protein-coding Sequences Inside “Non-coding” RNAs

**DOI:** 10.1016/j.gpb.2016.03.002

**Published:** 2016-04-09

**Authors:** Daniel Oehler, Jan Haas

**Affiliations:** 1 Institute for Cardiomyopathies, Department of Internal Medicine III, University of Heidelberg, 69120 Heidelberg, Germany; 2 German Centre for Cardiovascular Research (DZHK), Heidelberg/Mannheim, Germany

## Calcium homeostasis is crucial for muscle contractility

Muscle cells are critically dependent on calcium homeostasis. Without having the right amount of calcium ions just on the spot and coordinated in between muscle cells, no contraction can take place. Therefore, calcium homeostasis is one of the critical regulatory mechanisms in all muscle cells, including skeletal muscle and heart [1], [2]. Sarco-endoplasmic reticulum Ca^2+^ adenosine triphosphatase (SERCA) is responsible for the relaxation of muscle cells by pumping Ca^2+^ into the sarcoplasmic reticulum (SR) [3], [4]. For the process of muscle contraction, not only excitation via calcium-release but also the quick reestablishing of the baseline calcium gradient is absolutely necessary. Proteins known to be involved in this process include phospholamban (PLN; inhibition of SERCA in phosphorylated state) [5], [6], [7], sarcolipin (SLN; uncoupling of SERCA in heat regulation) [7], [8], [9], and myoregulin (MLN; inhibition of skeletal muscle SERCA) [10].

## DWORF – a peptide “hiding” in a non-coding RNA

Recently Nelson et al. published a paper in *Science* [11], describing their discovery of a so-far unknown small protein, named dwarf open reading frame (DWORF), which could function as an activator of SERCA [11]. DWORF consists of 34 amino acid residues and “is the third smallest full-length protein known to be encoded by the mouse genome” [11]. Astonishingly, DWORF is encoded by a locus within the open reading frame of a previously-annotated long non-coding RNA (lncRNA) gene (“NONMMUG026737” in mice, corresponding to “LOC100507537” in humans). Notably, a similar genomic structure is reported for the gene encoding MLN, which is located within a previously-annotated lncRNA named LINC00948 in humans [10].

To understand the impact of these findings, one must take a look at the background of lncRNAs. lncRNAs are commonly defined as RNAs that have more than 200 nucleotides in length and appear not to have coding potential [12]. The length distinguishes lncRNAs from small regulatory RNAs like microRNAs, while it is clear that there is not always a clear cut-off in discrimination to other RNA subtypes [13]. The biological function of lncRNAs — without going into more detail here — is not always easy to identify and seems to be very diverse [14], [15]. In the past years, many newly-described so-called “lncRNA” have been published and it seems that they have biological implication in animal models for different diseases, but their precise role mostly remains unclear [14], [15]. In the aforementioned paper, the authors searched explicitly for “masked” proteins inside areas previously described as non-coding regions using a recognition software such as PhyloCSF [16]. Doing so, they were able to detect protein-coding information hidden in RNAs, which literally had been “thrown away” before. This is of course not true for every lncRNA. As we understand too little about the functions of lncRNAs in general, sometimes misleading interpretations as described above can occur, making it crucial to understand and divide this heterogeneous group of RNAs for correctly analyzing their functional implications (*e.g.*, regulatory roles).

## DWORF – increasing contractility by improving relaxation

DWORF is conserved across species, which gives a glimpse at the importance of this protein. Though it is expressed in the heart and in slow-twitch muscle fibers, there is a lack of gene expression of *DWORF* in fast-twitch muscle. Slow twitch muscle cells contract slower and efficiently use oxygen for enduring contraction purposes, while in contrast fast-twitch muscle cells use preferably anaerobic pathways for short-term contractions [17]. In other words, the DWORF regulates muscle cells involved in long-term contractility such as the heart.

The authors confirmed the expression of the presumed protein (*e.g.*, by Western blotting) and showed that SERCA and DWORF not only co-localize, but also interact. In other words, DWORF activates SERCA using the same binding domain as PLN with similar binding affinities. In tetanic stimulated muscle cells, DWORF enhanced the relaxation time, which could not be seen with lower stimulation frequencies. Therefore, the authors conclude that DWORF might be a positive enhancer for re-compensation of muscle contractility after tetanic stimulation by fast “filling” the pool of Ca^2+^ in SR. This activation of SERCA is due to inhibition of PLN, which itself inhibits the SERCA-mediated calcium flow, conclusive to the finding that they act on the same binding site.

By performing further functional analysis, the authors showed that DWORF is an indirect activator of SERCA. With overexpression of DWORF, transgenic mice showed an increased influx into SR. Consecutively, the contractility in these mice was significantly higher due to a higher relaxation rate and higher load of Ca^2+^ inside SR.

From the physicians’ point of view, the question remains how this finding could be translated into novel therapies. In their paper, the authors show a downregulation of DWORF not only in the dilated cardiomyopathy (DCM) mice but also in failing human hearts [11]. Currently, the baseline therapy for patients with a failing heart is to minimize workload and protect it from pathological stress. Combining the findings of the paper, there are two possibilities taking benefit out of these: First, DWORF could be used as a new biomarker in DCM patients showing the loss of contractility and therefore the severity of the heart failure state in general. This could give physicians the opportunity to optimize therapy before the patient is showing a deteriorated clinical course. Second, DWORF can be considered as a new drug target. By activating or increasing the amount of DWORF in heart failing muscle cells, the contractility could be raised by activating SERCA. This would be a new therapeutic way, which would go hand in hand with the baseline therapy of patients with severe heart failure.

In summary, the search for proteins “hidden in the black space of the genome” will certainly lead to novel surprises as to the complexity of the human heart. Bioinformatics methods are the first stage in this exciting discovery process.

## Competing interests

The authors declare that they have no competing interests.
